# Tea Consumption and Risk of Head and Neck Cancer

**DOI:** 10.1371/journal.pone.0096507

**Published:** 2014-05-05

**Authors:** Cheng-Chih Huang, Wei-Ting Lee, Sen-Tien Tsai, Chun-Yen Ou, Hung-I Lo, Tung-Yiu Wong, Sheen-Yie Fang, Ken-Chung Chen, Jehn-Shyun Huang, Jiunn-Liang Wu, Chia-Jui Yen, Wei-Ting Hsueh, Yuan-Hua Wu, Ming-Wei Yang, Forn-Chia Lin, Jang-Yang Chang, Kwang-Yu Chang, Shang-Yin Wu, Jenn-Ren Hsiao, Chen-Lin Lin, Yi-Hui Wang, Ya-Ling Weng, Han-Chien Yang, Jeffrey S. Chang

**Affiliations:** 1 Department of Otolaryngology, National Cheng Kung University Hospital, College of Medicine, National Cheng Kung University, Tainan, Taiwan; 2 Department of Stomatology, National Cheng Kung University Hospital, College of Medicine, National Cheng Kung University, Tainan, Taiwan; 3 Division of Hematology/Oncology, Department of Internal Medicine, National Cheng Kung University Hospital, College of Medicine, National Cheng Kung University, Tainan, Taiwan; 4 Department of Radiation Oncology, National Cheng Kung University Hospital, College of Medicine, National Cheng Kung University, Tainan, Taiwan; 5 National Institute of Cancer Research, National Health Research Institutes, Tainan, Taiwan; 6 Department of Nursing, National Cheng Kung University Hospital, College of Medicine, National Cheng Kung University, Tainan, Taiwan; Universität Bochum, Germany

## Abstract

**Background:**

The current study evaluated the association between tea consumption and head and neck cancer (HNC) in Taiwan, where tea is a major agricultural product and a popular beverage.

**Methods:**

Interviews regarding tea consumption (frequency, duration, and types) were conducted with 396 HNC cases and 413 controls. Unconditional logistic regression was performed to estimate the odds ratio (OR) and 95% confidence interval (CI) of HNC risk associated with tea drinking, adjusted for sex, age, education, cigarette smoking, betel quid chewing, and alcohol drinking.

**Results:**

A reduced HNC risk associated with tea drinking (OR for every cup per day = 0.96, 95% CI: 0.93–0.99; OR for ≧5 cups per day = 0.60, 95% CI: 0.39–0.94) was observed. The association was especially significant for pharyngeal cancer (OR for every cup per day = 0.93, 95% CI: 0.88–0.98; OR for ≧5 cups per day = 0.32, 95% CI: 0.16–0.66). A significant inverse association between HNC and tea consumption was observed particularly for green tea.

**Conclusions:**

This study suggests that tea drinking may reduce the risk of HNC. The anticancer property of tea, if proven, may offer a natural chemopreventive measure to reduce the occurrence of HNC.

## Introduction

Head and neck cancer (HNC), including cancers of the oral cavity, pharynx (oropharynx and hypopharynx), and larynx, has an annual incidence of approximately 550,000 cases and is the seventh most common cancer in the world [1]. Alcohol drinking and consumption of tobacco products are two major risk factors of HNC. Approximately 70% of HNC cases can be attributed to the consumption of alcohol and tobacco [2]. In areas with a high prevalence of betel quid chewing, a large proportion of HNC cases is caused by chewing betel quid [2]. Another major risk factor for HNC is human papillomavirus (HPV) infection, particularly for oropharyngeal cancer [3]. In contrast to the well-established risk factors of HNC, little is known about factors that reduce the risk of HNC, with the most consistent finding being a lower risk of HNC associated with a higher consumption of fruits and vegetables [2].

Tea, which is produced from the leaves of *Camellia sinensis*, is the second most popular beverage in the world [4]. Depending on the degree of fermentation, tea can be divided into green tea (non-fermented), oolong tea (semi-fermented), and black tea (fully fermented) [4]. Black tea is most widely consumed in the United States, Europe and Western Asia and accounts for 78% of the worldwide tea production [5]. Green tea, which accounts for 20% of the worldwide tea production, is most often consumed in Japan and parts of China [5]. Two percent of the worldwide tea production is oolong tea, which is popular in Taiwan and southeastern China [5].

Tea contains an abundance of polyphenols. Catechins are the major polyphenols in green tea (30–42% of all polyphenols in green tea) and 50–65% of the catechins in green tea are epigallocatechin-3-gallate (EGCG) [6]. During the fermentation process to produce black tea, catechins are oxidized and polymerized to form theaflavins and thearubigins, which are the two major polyphenols of black tea [4], [6]. Compared to green tea, black tea contains much less catechins (3–10% of all polyphenols in black tea) [6]. In laboratory studies, tea polyphenols, particularly EGCG, have been shown to possess anti-tumor properties by inducing cell cycle arrest and apoptosis, and inhibiting angiogenesis, proliferation, invasion, and metastasis of cancer cells [6].

Although many epidemiologic studies have been conducted to investigate the association between tea drinking and HNC, the results have been inconsistent [7]–[26]. Many of these studies only perform a crude assessment of tea consumption without detailed information on the amount of tea drinking [9]–[12], [15], [16], [18], [20]. Among those studies that did assess the amount of tea drinking, the prevalence of tea consumption was often low and the range of the amount consumed was often too narrow, with some studies comparing those who drank 1 cup/day to non-drinkers. In addition, only a few studies examined this association by the types of tea [12], [15], [19], [26].

The current study evaluated the association between tea consumption and HNC in Taiwan, where tea is a major agricultural product and a popular beverage. Detailed information on the frequency and duration of tea drinking and the types of tea was included. In addition, we investigated whether tea drinking can interact with consumption of alcohol, betel quid, or cigarette to influence the risk of HNC.

## Materials and Methods

### Ethic statement

This study was approved by the Institutional Review Board of the National Cheng Kung University Hospital and the Research Ethics Committee of National Health Research Institutes. Every participant signed an informed consent.

### Study subjects

All study subjects (HNC cases and controls) were recruited from the Department of Otolaryngology and the Department of Stomatology at the National Cheng Kung University Hospital from September 1, 2010 to May 30, 2013. Cases were patients newly diagnosed with pathologically confirmed squamous cell carcinoma of the oral cavity, pharynx (oropharynx and hypopharynx), and larynx, who had no previous diagnosis of cancer, and aged 20 to 80 years. Controls were patients who underwent surgery for non-cancerous diseases unrelated to the use of alcohol, betel quid, and cigarette, and had no previous diagnosis of cancer. In addition, those with pre-cancerous lesions (leukoplakia and erythroplakia) of the oral cavity, oropharynx, hypopharynx, and larynx were excluded. Controls were frequency-matched to the cases on age (±5 years) and sex.

### Data collection

A trained interviewer interviewed each study participant about the consumption of tea. Questions on tea drinking included the type of tea (green tea, oolong tea, or black tea) and the frequency of tea consumption (never, occasional, or daily). For those who drank tea daily, more questions were asked separately for each type of tea regarding the number of cups (1 cup  =  150 ml) per day and the total years of tea drinking. Because the question on the total years of tea drinking was added later in the study, 33 cases (8.3%) and 30 controls (7.3%) did not have this information. Additional data were collected to adjust for potential confounders, including demographic factors (sex, age, and educational level), use of alcohol, betel quid, and cigarette, and consumption of vegetables and fruits.

### Statistical analysis

To compare the distributions of demographic variables and lifestyle habits (alcohol drinking, betel quid chewing, cigarette smoking, and consumption of vegetables and fruits) between cases and controls, t-tests (for continuous variables) and chi-squared tests (for categorical variables) were performed.

Multivariable unconditional logistic regression was performed to estimate the odds ratio (OR) and 95% confidence interval (CI) of HNC associated with tea drinking adjusted for age, sex, education, alcohol drinking (frequency), betel quid use (pack-years), and cigarette smoking (pack-years). Age was adjusted as a continuous variable. Education was adjusted as an ordinal variable (1  =  ≦ elementary school; 2  =  junior high; 3  =  high school/technical school; and 4  =  some college or more). Alcohol drinking was adjusted by using the frequency of alcohol drinking as an ordinal variable (1  =  never; 2  =  1-2 drinks per week; 3  =  3–5 drinks per week; and 4  =  daily drinkers). Cigarette smoking and betel quid chewing were adjusted as continuous variables in pack-years. One pack-year of cigarette smoking  =  1 pack of cigarette (20 cigarettes) use per day ×1 year. One pack year of betel quid use  =  1 pack of betel quid (20 betel quids) use per day ×1 year. Since adjusting for the intake of vegetables and fruits changed the OR by less than 10%, these two variables were not retained in the final statistical models. The association between HNC risk and tea drinking was analyzed in several ways: 1) by the number of cups per day as a continuous variable or categorized as never, occasional, or daily. For never and occasional tea drinkers, the number of cups per day  =  0; 2) by the total years of tea drinking; and 3) by the total cup-year of tea drinking, with 1 cup-year  =  1 cup per day ×1 year. The linearity assumption for the number of cups per day, total years of tea drinking, or the total cup-year was checked with the restricted cubic spline function using the %RCS_REG SAS macro written by Desquilbet and Mariotti [27]. The results indicated no evidence of non-linearity for these variables. The analysis was first performed with all HNC combined and then by sites (oral cavity, pharynx, and larynx). Additional analyses were performed for the different types of tea (green tea, oolong tea, and black tea).

To assess the effect modification of alcohol, betel quid or cigarette on the association between tea drinking and HNC risk, unconditional logistic regression was performed stratified on the use of alcohol, betel quid, or cigarette. The heterogeneity between strata was tested on the multiplicative scale by comparing the full regression model with an interaction term (tea × alcohol, tea × betel quid, or tea × cigarette) to the sub-model without the interaction term using the log-likelihood ratio test with 1 degree of freedom.

Lastly, we evaluated whether tea drinking is associated with a less aggressive behavior of HNC by examining whether tea drinking is associated with HNC of early stages, lower tumor grade, and older onset. Information on stage and tumor grade was obtained from the cancer registry of the National Cheng Kung Hospital. Pathological stage was used for HNC patients who underwent surgery while clinical stage was used for those who received other types of therapies. Stage and grade were not available for 35 (10%) and 138 (35%) of HNC patients, respectively. Those with missing data were not significantly different from those with non-missing data in the distributions of age, sex, and the consumption of alcohol, betel quid, and cigarette.

## Results

From September 1, 2010 to May 30, 2013, 396 HNC cases (262 oral cancers, 96 oro- and hypopharyngeal cancers, and 38 laryngeal cancers) and 413 controls were successfully recruited with a participation of 78% and 89%, respectively. The 413 controls consisted of patients with various benign otolaryngeal conditions, including benign laryngeal lesions (n = 15), benign oral lesions (n = 16), benign pharyngeal lesions (n = 7), benign salivary gland tumor (n = 60), cheek abscess (n = 1), cheek cyst (n = 6), cholesteatoma (n = 1), chronic otitis media (n = 17), chronic rhinitis (n = 12), chronic sinusitis (n = 56), epiglottic cyst (n = 8), epistaxis (n = 2), esophageal stenosis (n = 1), external auditory canal osteoma (n = 1), incomplete glottic closure (n = 1), laryngeal cyst (n = 1), laryngocele (n = 1), mastoiditis (n = 1), middle turbinate headache syndrome (n = 2), nasal polyp (n = 7), nasal septum deviation (n = 2), nasal synechiae (n = 1), neck abscess (n = 4), neck lipoma (n = 26), obstructive sleep apnea (n = 8), sialolithiasis (n = 11), thyroglossal duct cyst (n = 8), tonsillitis (n = 11), vocal cord cyst (n = 9), vocal cord granuloma (n = 4), vocal cord nodules (n = 56), vocal cord palsy (n = 4), vocal cord polyp (n = 52), and wegener's granulomatosis (n = 1). HNC cases and controls were similar in the distributions of age and sex (*P*>0.05) (Table 1). Controls had higher levels of education than cases (*P*<0.0001) with more controls having at least a high school education (62.7% vs. 39.4%). HNC cases consumed more alcohol, betel quid, and cigarette than controls (*P*<0.0001).

**Table 1:** Demographic and lifestyle characteristics of the head and neck cancer patients and control subjects.

Characteristics	Cases	Controls	P-value^a^
	N = 396	N = 413	
	n (%)	n (%)	
**Age (years)**			
Mean (SE)	54.6 (0.5)	53.9 (0.5)	0.34
**Sex**			
Male	374 (94.4)	391 (94.7)	0.89
Female	22 (5.6)	22 (5.3)	
**Education**			
≦ Elementary school	120 (30.3)	72 (17.4)	<0.0001
Junior high	120 (30.3)	82 (19.9)	
High school/Technical school	122 (30.8)	150 (36.3)	
Some college or more	34 (8.6)	109 (26.4)	
**Alcohol**			
Never + occasional	116 (29.3)	216 (52.3)	<0.0001
Former regular	68 (17.2)	52 (12.6)	
Current regular	212 (53.5)	145 (35.1)	
			
Never	100 (25.3)	178 (43.1)	<0.0001
1 drink per month	16 (4.0)	38 (9.2)	
1–2 drinks per week	13 (3.3)	27 (6.5)	
3–5 drinks per week	28 (7.1)	39 (9.4)	
Daily drinkers	229 (57.8)	127 (30.8)	
Unknown	10 (2.5)	4 (1.0)	
**Betel quid**			
Never	121 (30.6)	311 (75.3)	<0.0001
Former	179 (45.2)	67 (16.2)	
Current	96 (24.2)	35 (8.5)	
Never	121 (30.5)	311 (75.4)	<0.0001
0.1–9.9 pack-years	50 (12.6)	34 (8.2)	
10.0–19.9 pack-years	47 (11.9)	15 (3.6)	
20.0–29.9 pack-years	38 (9.6)	14 (3.4)	
30.0 or more pack-years	123 (31.1)	38 (9.2)	
Unknown	17 (4.3)	1 (0.2)	
Pack-years (SE)	26.5 (2.1)	7.1 (1.0)	<0.0001
**Cigarette**			
Never	48 (12.2)	124 (30.1)	<0.0001
Former	86 (21.7)	93 (22.6)	
Current	261 (66.1)	195 (47.3)	
			
**Never**	48 (12.1)	124 (30.0)	<0.0001
0.1–9.9 pack-years	21 (5.3)	34 (8.2)	
10.0–19.9 pack-years	47 (11.9)	56 (13.6)	
20.0–29.9 pack-years	66 (16.7)	58 (14.0)	
30.0 or more pack-years	207 (52.2)	136 (33.0)	
Unknown	7 (1.8)	5 (1.2)	
			
Pack-years (SE)	34.9 (1.3)	24.0 (1.3)	<0.0001
**Vegetable intake**			
Non-daily	82 (20.8)	45 (10.9)	0.0001
Daily	312 (79.2)	368 (89.1)	
**Fruit intake**			
Non-daily	284 (72.3)	209 (50.6)	<0.0001
Daily	109 (27.7)	(49.4)	

Abbreviations: N  =  number; SE  = standard error.

a. P-values were calculated using chi-squared tests (for categorical variables) or T-tests (for continuous variables).

Compared to never tea drinkers, daily tea drinking was associated with a reduced risk of HNC (OR = 0.66, 95% CI: 0.44–0.98), especially for those who drank 5 or more cups per day (OR = 0.60, 95% CI: 0.39–0.94) (Table 2). The reduction of cancer risk associated with tea drinking was especially prominent for pharyngeal cancer (For daily drinkers: OR = 0.35, 95% CI: 0.19–0.67; for 5 or more cups per day: OR = 0.32, 95% CI: 0.16–0.66). Every cup of tea drinking per day was associated with a 4% reduction in the risk of HNC (OR = 0.96, 95% CI: 0.93–0.99), a 4% reduction in the risk of oral cancer (OR = 0.96, 95% CI: 0.93–1.00), and a 7% reduction in the risk of pharyngeal cancer (OR = 0.93, 95% CI: 0.88–0.98); however, no significant association with laryngeal cancer was observed. Although every year of tea drinking and every 10 cup-year of tea drinking were associated with a reduced risk of HNC, none of these associations was statistically significant.

**Table 2:** Tea consumption and head and neck cancer risk, overall and by sites.

Tea consumption	Controls	All cases	OR (95% CI)^a^	Oral cancer	OR (95% CI)^a^	Pharyngeal cancer	OR (95% CI)^a^	Laryngeal cancer	OR (95% CI)^a^
	N = 413	N = 396		N = 262		N = 96		N = 38	
	n (%)	n (%)		n (%)		n (%)		n (%)	
Never	109 (26.4)	134 (33.8)	Referent	78 (29.8)	Referent	42 (43.7)	Referent	14 (36.8)	Referent
Occasional	105 (25.4)	92 (23.2)	0.84 (0.54–1.32)	66 (25.2)	1.05 (0.62–1.76)	21 (21.9)	0.52 (0.25–1.07)	5 (13.2)	0.47 (0.15–1.47)
Daily	199 (48.2)	170 (42.9)	0.66 (0.44–0.98)	118 (45.0)	0.75 (0.46–1.17)	33 (34.4)	0.35 (0.19–0.67)	19 (50.0)	0.65 (0.28–1.53)
1–4 cups/day	72 (17.4)	59 (14.9)	0.77 (0.46–1.29)	35 (13.4)	0.80 (0.44–1.48)	14 (14.6)	0.45 (0.20–1.02)	10 (26.3)	0.89 (0.32–2.47)
≧5 cups/day	124 (30.1)	108 (27.2)	0.60 (0.39–0.94)	80 (30.5)	0.69 (0.42–1.16)	19 (19.8)	0.32 (0.16–0.66)	9 (23.7)	0.54 (0.20–1.45)
Missing	3 (0.7)	3 (0.8)	-	3 (1.1)	-	0 (0.0)	-	0 (0.0)	-
P for trend			0.02		0.11		0.002		0.35
Every cup of tea per day^b^			0.96 (0.93–0.99)		0.96 (0.93–1.00)		0.93 (0.88–0.98)		0.96 (0.89–1.05)
Every year of tea drinking^b^			0.99 (0.97–1.01)		0.99 (0.97–1.01)		0.98 (0.96–1.01)		0.98 (0.95–1.02)
Every 10 cup-year of tea drinking^b,c^			0.99 (0.97–1.01)		0.99 (0.98–1.01)		0.98 (0.96–1.01)		0.97 (0.91–1.02)

Abbreviations: CI  =  confidence interval; N  =  number; OR  =  odds ratio.

a. OR and 95% CI were calculated using unconditional logistic regression, adjusted for sex, age, education, cigarette smoking (pack-year categories), betel quid chewing (pack-year categories), and alcohol drinking (frequency).

b. For never and occasional tea drinkers, the cup of tea per day  =  0, the year of tea drinking = 0, and the cup-year of tea drinking = 0.

c. 1 cup-year  =  1 cup of tea per day ×1 year.

Table 3 presents the association between different types of tea and HNC risk. The inverse association between tea and HNC risk appeared the strongest for green tea, with a 6% reduction in HNC risk associated with every cup of green tea per day (OR = 0.94, 95% CI: 0.90–0.98). Every cup of oolong tea was associated with a 4% reduction in HNC risk, although not statistically significant (OR = 0.96, 95% CI: 0.91–1.01). Drinking black tea was not significantly associated with the risk of HNC; however it appeared that daily drinking of black tea, especially 3 or more cups per day, was associated with a non-significantly reduced risk of HNC (OR = 0.59, 95% CI: 0.26–1.33).

**Table 3:** Tea consumption and head and neck cancer risk by the types of tea.

Tea consumption	Cases	Controls	OR (95% CI)^a^
	N = 396	N = 413	
	n (%)	n (%)	
**Green Tea**			
Never	217 (54.8)	213 (51.7)	Referent
Occasional	97 (24.5)	98 (23.8)	1.02 (0.65–1.61)
Daily	82 (20.7)	101 (24.5)	0.62 (0.39–0.99)
1–2 Cups Daily	24 (6.1)	22 (5.3)	1.37 (0.63–2.99)
3 or more Cups Daily	57 (14.4)	78 (19.0)	0.49 (0.29–0.83)
Missing	1 (0.2)	1 (0.2)	-
P for trend			0.01
Every cup per day			0.94 (0.90–0.98)
**Oolong Tea**			
Never	197 (49.8)	204 (49.4)	Referent
Occasional	107 (27.0)	111 (26.8)	0.91 (0.58–1.41)
Daily	92 (23.2)	98 (23.7)	0.75 (0.48–1.20)
1–2 Cups Daily	29 (7.3)	35 (8.5)	0.77 (0.40–1.48)
3 or more Cups Daily	61 (15.4)	63 (15.2)	0.72 (0.43–1.21)
P for trend			0.19
Every cup per day			0.96 (0.91–1.01)
**Black Tea**			
Never	258 (65.1)	257 (62.2)	Referent
Occasional	112 (28.3)	128 (31.0)	0.76 (0.49–1.17)
Daily	26 (6.6)	28 (6.8)	0.70 (0.35–1.43)
1–2 Cups Daily	8 (2.0)	8 (1.9)	1.10 (0.33–3.72)
3 or more Cups Daily	18 (4.6)	20 (4.9)	0.59 (0.26–1.33)
P for trend			0.18
Every cup per day			1.00 (0.90–1.11)

Abbreviations: CI  =  confidence interval; N  =  number; OR  =  odds ratio.

a. OR and 95% CI were calculated using unconditional logistic regression, adjusted for sex, age, education, cigarette smoking (pack-year categories), betel quid chewing (pack-year categories), alcohol drinking (frequency), and drinking of other types of tea

Table 4 presents the results of the analyses on the effect modification of the relationship between tea drinking and HNC risk by alcohol, betel quid, or cigarette. Tea drinking appeared to reduce the risk of HNC among regular alcohol drinkers but not among never or occasional alcohol drinkers. These were observed for every cup of tea per day (*P* for interaction = 0.007) and for every 10 cup-year of tea drinking (*P* for interaction = 0.03). Among regular alcohol drinkers, every cup of tea per day and every 10 cup-year of tea drinking was associated with a decreased risk of HNC among daily alcohol drinkers but not among non-daily alcohol drinkers (*P* for interaction =  0.07 and 0.06, respectively). No effect modification on the association between tea drinking and HNC risk by betel quid chewing or cigarette smoking was observed.

**Table 4:** The association between tea drinking and risk of head and neck cancer stratified by the use of alcohol, betel quid, or cigarette.

	5 or more cups daily vs. never + < 5 cups daily	Every cup of tea per day^b^	Every 10 cup-year of tea drinking^b,c^
	OR (95% CI)^a^	OR (95% CI)^a^	OR (95% CI)^a^
**Alcohol drinking**			
Never + occasional	1.03 (0.56–1.90)	1.01 (0.97–1.06)	1.01 (0.99–1.03)
Former regular	0.47 (0.17–1.31)	0.93 (0.86–1.00)	0.97 (0.94–1.01)
Current regular	0.57 (0.32–1.01)	0.93 (0.88–0.98)	0.98 (0.95–1.00)
Former regular + current regular	0.55 (0.34–0.89)	0.93 (0.89–0.97)	0.98 (0.96–1.00)
	P-interaction = 0.11^e^	P-interaction = 0.007^e^	P-interaction = 0.03^e^
Regular drinkers only^d^			
< 28 years	0.46 (0.23–0.95)	0.91 (0.86–0.97)	0.98 (0.95–1.01)
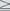 28 years	0.66 (0.33–1.31)	0.93 (0.87–1.00)	0.98 (0.94–1.01)
	P-interaction = 0.82	P-interaction = 0.98	P-interaction = 0.97
Non-daily drinkers	1.38 (0.41–4.66)	1.03 (0.93–1.15)	1.02 (0.98–1.07)
Daily drinkers	0.52 (0.30–0.89)	0.92 (0.88–0.97)	0.97 (0.95–0.99)
	P-interaction = 0.27	P-interaction = 0.07	P-interaction = 0.06
**Betel quid**			
Never	0.65 (0.37–1.13)	0.98 (0.94–1.03)	1.00 (0.98–1.02)
Former	0.73 (0.39–1.38)	0.95 (0.91–1.00)	0.99 (0.97–1.02)
Current	0.90 (0.37–2.19)	0.97 (0.90–1.04)	0.98 (0.95–1.01)
Former + Current	0.71 (0.43–1.16)	0.95 (0.91–0.99)	0.99 (0.97–1.01)
	P-interaction = 0.96^e^	P-interaction = 0.15^e^	P-interaction = 0.18^e^
Betel quid chewer only^d^			
< 21 pack-years	0.69 (0.32–1.50)	0.94 (0.88–1.01)	0.98 (0.93–1.02)
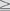 21 pack-years	0.76 (0.37–1.58)	0.95 (0.90–1.01)	0.99 (0.96–1.01)
	P-interaction = 0.97	P-interaction = 0.95	P-interaction = 0.95
**Cigarette**			
Never	0.32 (0.08–1.34)	0.94 (0.81–1.08)	1.00 (0.90–1.11)
Former	1.28 (0.58–2.84)	1.00 (0.92–1.08)	1.02 (0.98–1.07)
Current	0.64 (0.40–1.01)	0.96 (0.92–1.00)	0.99 (0.97–1.00)
Former + Current	0.75 (0.51–1.11)	0.97 (0.94–1.00)	0.99 (0.98–1.01)
	P-interaction = 0.35^e^	P-interaction = 0.80^e^	P-interaction = 0.92^e^
Cigarette smokers only^d^			
< 28 pack-years	0.93 (0.48–1.80)	0.95 (0.89–1.01)	0.96 (0.93–1.00)
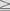 28 pack-years	0.64 (0.39–1.05)	0.97 (0.93–1.01)	1.00 (0.98–1.02)
	P-interaction = 0.23	P-interaction = 0.99	P-interaction = 0.27

Abbreviations: CI  =  confidence interval; OR  =  odds ratio

a. OR and 95% CI were calculated using unconditional logistic regression, adjusted for sex, age, education, cigarette smoking (pack-year categories), betel quid chewing (pack-year categories), and alcohol drinking (frequency)

b. For never and occasional tea drinkers, the cup of tea per day  =  0 and the cup-year of tea = 0.

c. 1 cup-year  =  1 cup of tea per day ×1 year

d. Years of consumption was cut off at the median

e. Tests for interaction were performed using only two strata (Never and former+current).

Table 5 presents the distributions of HNC stage, grade, and age of diagnosis according to the status of tea drinking (never + occasional vs. daily). HNC patients who were daily drinkers of tea tended to be diagnosed at an earlier stage than those who were never or occasional tea drinkers, although the difference was not statistically significant. The distributions of tumor grade and age of diagnosis were not different by tea drinking status. Analyses with green tea also showed that daily drinkers had more HNCs diagnosed at early stages, although the result was also not statistically significant.

**Table 5:** The stage, tumor grade, and mean age of diagnosis of HNC according to tea drinking status.

	Any type of tea			Green Tea		
	Never or occasional drinkers	Daily drinkers	P-value^a^	Never or occasional drinkers	Daily drinkers	P-value^a^
	N (%)	N (%)		N (%)	N (%)	
**Stage**						
1+2	78 (38.2)	75 (47.8)	0.07	115 (40.2)	38 (50.7)	0.10
3+4	126 (61.8)	82 (52.2)		171 (59.8)	37 (49.3)	
**Tumor grade**						
Well differentiated	42 (37.8)	53 (36.1)	0.96	68 (34.7)	27 (43.6)	0.35
Moderately differentiated	58 (52.3)	79 (53.7)		109 (55.6)	28 (45.2)	
Poorly differentiated	11 (9.9)	15 (10.02)		19 (9.7)	7 (11.3)	
**Age at diagnosis**						
Mean (SE)	54.8 (0.7)	54.2 (0.8)	0.58	54.7 (0.6)	54.1 (1.2)	0.63

Abbreviations: SE standard error

a. P-values were calculated using chi-squared tests (for categorical variables) or T-tests (for continuous variables).

We repeated the entire analysis with men only and the results were similar.

## Discussion

In this study, we observed a reduced risk of HNC associated with tea drinking. This association was especially significant for pharyngeal cancer, although it was also present for oral cancer. An inverse association between HNC and tea consumption was observed for green tea and oolong tea, although it was statistically significant only for green tea. Finally, the reduced risk of HNC associated with tea drinking was observed only among regular alcohol drinkers but not among occasional or never drinkers.

The significant inverse association between tea drinking and HNC was reported by 5 published studies [10], [21], [22], [25], [26], while 3 reported a non-significant inverse association [11], [14], [19], 11 reported no association [7]–[9], [12], [13], [15]–, and 1 reported a significant positive association [18]. Several factors may explain the inconsistencies between studies. First, many studies only crudely assessed the consumption of tea with insufficient information on the amount consumed. Second, most of the studies were conducted in Europe and America, where the prevalence of tea consumption was often not high, and among the tea drinkers, the amount of tea consumed was often low. In our study, about 75% of controls drank tea and nearly 50% of them were daily tea drinkers with 30% drinking 5 (750 ml) or more cups of tea per day. Thus, compared to most of the other studies, our study had both a high prevalence and a sufficient range of tea consumption to better assess the association between tea drinking and HNC. Finally, studies conducted in Europe and America most likely assessed the consumption of black tea, whereas the most commonly consumed tea in our study population is green tea and oolong tea. If the types of tea matter in the association between tea and HNC, it may explain part of the inconsistencies between studies.

In our study, a reduced risk of HNC risk was significantly associated with green tea and non-statistically associated with oolong tea and black tea (for those who consumed 3 or more cups per day). Only four published studies have assessed the association between HNC risk and green tea. In a hospital-based case-control (404 cases and 404 controls) study from China, Zheng *et al.* reported a non-significantly reduced risk of oral cancer associated with green tea drinking (OR = 0.85, 95% CI: 0.32–2.31) [12]. However, the green tea consumption of that study was low with only 7.4% of study subjects drinking 1 or more cups of green tea per month [12]. In a cohort study of 7,995 Japanese American men, Chyou *et al.* reported no association between green tea and risk of upper aerodigestive tract (UADT) cancer (HNC + esophageal cancer) (relative risk: 1.14, 95% CI: 0.68–1.91) [15]. However, that study only compared ever green tea drinkers to never drinkers and in addition, the small number (n = 92) of incident UADT cancer cases likely provided insufficient statistical power [15], which we calculated to be 0.32 for detecting a relative risk of 0.67 or 1.5 [28]. In a cohort study of 20,550 men and 29,671 women from Japan, Ide *et al.* reported a non-significantly reduced risk of oral cancer associated with green tea consumption (*P* for trend = 0.07) [19]. Again, due to the small number (n = 37) of incident HNC cases, the study by Ide et al. was underpowered [19]. In a case-control study from China with 723 oral cancer cases and 857 controls by Fu *et al.*, black tea was not associated with the risk of oral cancer and green tea was associated with a reduced risk of oral cancer only among men [26]. The results of the study by Fu *et al.* are consistent with our results, because approximately 95% of our study subjects are men. Even though we did not find a statistically significant association between black tea and HNC, the association between black tea and HNC could not be ruled out, because only 7% of our study subjects were daily drinkers of black tea. In addition, our study showed that drinking 3 or more cups of black tea per day was associated with a non-significantly reduced risk of HNC (OR = 0.59, 95% CI: 0.26–1.33). Again, due to the small proportion (5%) of subjects who reported drinking 3 or more cups of black tea, the non-statistical significance could be due to insufficient statistical power (0.30 for detecting an OR = 0.59). More investigations are required to determine whether the association between tea consumption and HNC risk depends on the type of tea.

Our results showed that the inverse association between tea consumption and HNC is particular strong for pharyngeal cancers. Our results are consistent with two [14], [22] of the five published studies that examined the association between tea drinking and HNC by sites [7], [14], . The inconsistencies between studies and the relatively small sample size of pharyngeal cancer cases (n  =  96) in our study indicated that the particular strong inverse association between tea consumption and pharyngeal cancer in our study could be spurious. More investigations are needed to confirm our site-specific finding.

Our analysis showed an effect modification of alcohol drinking status but not cigarette smoking or betel quid chewing on the association between tea consumption and HNC risk. This suggested that tea consumption may be particularly effective in preventing the occurrence of HNC due to alcohol consumption, although the underlying biological mechanism is yet to be elucidated. The only other study that evaluated the interaction between alcohol consumption and tea drinking on the risk of HNC did not observe a difference in the association between tea drinking and HNC by alcohol consumption status [26]. Since our study is the first to show an effect modification of alcohol drinking status on the association between tea drinking and HNC, more studies are needed to confirm our finding. In addition, due to the multiple testing of 21 interaction tests (Table 4), chance finding could not be ruled out.

Most laboratory studies on the cancer chemopreventive activities of tea have been conducted on green tea polyphenols, particularly EGCG. These studies showed that EGCG exerts its cancer chemopreventive activities by targeting several signaling pathways at different cellular levels, including tyrosine kinase receptors (e.g. EGFR, VEGFR) at the cellular membrane, signaling molecules in cytoplasm (e.g. Akt), and transcription factors (e.g. NF-κB) [29]. The modulation of these signaling pathways by EGCG prevents oncogenesis by inducing apoptosis and cell cycle arrest and inhibiting angiogenesis and metastasis [29].

Results from clinical trials of patients with pre-malignant oral lesions support the chemoprevention of tea against oral cancer. In a double-blind randomized intervention trial with 59 patients with oral leukoplakia by Li *et al.*, 29 subjects received mixed tea (mixture of green tea extract, green tea polyphenols, and tea pigments) and 30 subjects received placebo [30]. After six months of trial, the size of oral lesions decreased in 38% of patients in the mixed tea group compared to 10% in the placebo group [30]. In addition, the incidence of micronuclei (indication for DNA damage) in the exfoliated oral mucosal cells and markers of cell proliferation were lower in the mixed tea group than the placebo group [30]. In another clinical trial of patients with oral premalignant lesions, patients were randomized to receive three different dosages of green tea extract or placebo for 12 weeks [31]. Fifty percent of those in the treatment group showed a complete response or partial response vs. 18% in the placebo group [31]. In addition, the clinical response showed a dose-response effect [31]. Biomarker analysis suggested that green tea extract may exert its effect on oral pre-malignant lesions by inhibiting angiogenesis [31]. More clinical trials are needed to assess and confirm the efficacy of tea and determine the best formulation and dosage to prevent oral cancer and other types of HNC.

Several limitations must be noted when interpreting the results of this study. In a hospital-based case-control study, it is often difficult to determine whether the cases and controls arise from the same source population. However, we have information to reasonably believe that this did not affect the results of our study. First, our study subjects were recruited from a major medical center located in Tainan City in southern Taiwan. More than 95% of our study subjects were residents of Tainan City and the surrounding Tainan County. In addition, efforts were made to exclude controls with diseases related to the use of alcohol, betel quid, and cigarette. Among our male controls, the percentages of ever alcohol drinkers and current betel quid chewers were 56.9% and 8.5%, respectively, which were consistent with those reported by a population-based survey of men living Tainan City and Tainan County (alcohol drinkers: 57.6%–65.6% and current betel quid chewers: 7.7%–15.4% [32]. Among our male controls, the percentage of current smokers (47.3%) was higher than that of male residents in Tainan City and Tainan County (28.0% to 33%) [32]. In Asian populations, cigarette smokers tend to drink more tea [5]. This is also true in our study population (among our controls: 56.3% of daily tea drinker among ever smokers vs. 29.8% of daily tea drinkers among never smokers). As a result, the higher percentage of current cigarette smokers in our controls than that of the general population could have biased our results towards the null. The true inverse association between tea consumption and HNC should have been stronger. Non-differential random recall error between cases and controls could also have biased our results towards the null. However, tea drinking is part of many Taiwanese' daily life and the regularity of tea consumption reduces the degree of recall error. In addition, previous studies showed that tea is a food item that can be recalled with high accuracy and reproducibility [33], [34]. Differential recall between cases and controls could also be possible, but this pertains more to the spurious positive association resulting from cases' being more likely to remember potentially harmful exposures. Lastly, we did not obtain information regarding the temperature of the tea. Previous studies suggested that high-temperature beverages may increase the risk of esophageal cancer. Whether high-temperature tea may influence the risk of HNC requires further investigation [35].

In summary, the current study showed an inverse association between HNC risk and tea consumption, particularly for green tea. In addition, the association between HNC risk and tea appeared to be modified by alcohol drinking status. Large cohort studies in populations with high tea consumption are needed to more clearly establish the beneficial effect of tea consumption in HNC prevention. Further evidence may also come from additional clinical trials with tea or tea extracts. Finally, more researches are needed to increase our understanding of the chemical constituents of tea and their biological activities. The anticancer property of tea, if proven, may offer a natural chemopreventive measure to reduce the occurrence of HNC.
